# A Smart Wheeled Walker With Integrated Physiological Monitoring to Improve Mobility in Older Adults: Randomized Crossover Trial

**DOI:** 10.2196/99522

**Published:** 2026-08-28

**Authors:** Phurichaya Werasirirat, Pornpimol Muanjai, Oranat Sukkho, Nongnuch Luangpon, Audrius Snieckus, Juntip Namsawang

**Affiliations:** 1 Department of Physical Therapy, Faculty of Allied Health Sciences, Burapha University, 169 Longhard Bangsaen Road, Muang District, Saen Suk, Chonburi, 20131, Thailand, 66 18788842; 2 Institute of Sport Science and Innovations, Lithuanian Sports University, Kaunas, Kaunas, Lithuania

**Keywords:** older adults, smart wheeled walker, assistive technology, Physiological Cost Index, dynamic balance

## Abstract

**Background:**

Mobility limitations in older adults are associated with an increased physiological cost of walking, reduced functional independence, and elevated fall risk. Conventional wheeled walkers improve stability but lack real-time physiological monitoring and safety feedback. Digital health–enabled assistive devices integrating physiological monitoring and fall detection may enhance mobility performance and safety.

**Objective:**

This study aimed to evaluate the short-term effects of a smart wheeled walker equipped with real-time physiological monitoring and fall detection compared with a standard wheeled walker in improving walking efficiency, dynamic balance, and fear of falling among community-dwelling older adults.

**Methods:**

A single-blind randomized crossover trial was conducted in 30 community-dwelling older adults aged 65 to 80 years with mild balance impairment (Timed Up and Go >13.5 seconds). Participants completed walking trials using both a standard wheeled walker and a smart wheeled walker in a randomized order, with a 30-minute washout period. The smart walker integrated photoplethysmography-based heart rate (HR) monitoring, pulse oximetry (oxygen saturation [SpO_₂_]), and a tilt-based fall detection system with mobile alert functionality. The primary outcome was walking efficiency assessed using the Physiological Cost Index (PCI). Secondary outcomes included dynamic balance (Expanded Timed Up and Go [ETUG]) and fear of falling (Falls Efficacy Scale-International). Statistical analysis was performed using paired comparisons with a significance level of *P*<.05.

**Results:**

Thirty (100%) participants completed both intervention conditions and were included in the final analysis. Walking speed was significantly higher with the smart wheeled walker than the standard wheeled walker (mean 28.77, SD 11.94 vs mean 18.14, SD 14.55 m/min; mean difference −10.63 m/min, 95% CI −17.51 to −3.75 m/min; *P*<.001). The PCI was significantly lower with the smart wheeled walker (mean 0.36, SD 0.38 vs mean 0.69, SD 1.23 beats/m; mean difference 0.33 beats/m, 95% CI 0.06‐0.60 beats/m; *P*=.02), indicating improved walking efficiency. Participants also completed the ETUG test significantly faster with the smart wheeled walker (mean 50.06, SD 24.50 vs mean 64.47, SD 23.04 s; mean difference 14.41 s, 95% CI 2.12‐26.70 s; *P*=.048), indicating improved dynamic balance. Fear of falling did not differ significantly between walker conditions (mean 31.56, SD 9.58 vs mean 32.91, SD 10.37; mean difference 1.35, 95% CI −3.81 to 6.51; *P*=.62).

**Conclusions:**

A smart wheeled walker integrating real-time physiological monitoring and fall detection significantly improved walking efficiency and dynamic balance during short-term supervised testing in community-dwelling older adults. These findings support the potential clinical value of digital health–enabled mobility aids. Further longitudinal studies conducted in real-world settings are warranted to evaluate long-term mobility, fall prevention, independent use, and comprehensive device safety.

## Introduction

Over the past decade, rapid global demographic aging has posed significant challenges to public health systems. By 2050, the global population aged 60 years and older is projected to reach approximately 2.1 billion, marking a dramatic rise in demand for older adult care and preventive strategies. Mobility impairments and balance disorders are key contributors to fall risk in older adults, with nearly one-third of community-dwelling seniors experiencing at least 1 fall annually—a proportion that increases to approximately 50% for those aged over 80 years [1,2]. Falls among older adults not only precipitate functional decline and loss of independence but are also a leading cause of injury-related mortality in this population [3].

In addition to biomechanical limitations, physiological responses during ambulation, such as elevated heart rate (HR) and increased oxygen consumption, have been shown to rise significantly among older adults with impaired mobility [4]. These responses reflect greater cardiovascular and muscular effort required for walking, which may contribute to fatigue and instability. A higher physiological cost of walking has also been associated with reduced gait efficiency and a greater risk of falls, particularly among frail individuals. Real-time monitoring of HR and oxygen saturation (SpO_₂_) during the use of a walker may thus serve as a meaningful indicator of exertion and functional capacity, enabling targeted interventions to optimize mobility assistance and reduce physiological strain [5]. Among current clinical strategies, the use of mobility aids such as wheeled walkers has proven effective in enhancing gait stability and reducing musculoskeletal burden [6,7]. Older adults using wheeled walkers exhibited significantly improved backward walking performance, including higher speed, greater step frequency, and fewer steps, indicating better motor control and energy efficiency [8]. The type of walking aid used can influence walking performance and physiological energy cost in older adults. These findings reinforce the importance of wheeled walkers in supporting older adults with compromised mobility [9].

Despite their benefits, traditional wheeled walkers have notable limitations related to maneuverability, brake use, and navigation in challenging situations, such as negotiating obstacles, slopes, and doorways [10,11]. Two-wheeled walkers were associated with a higher mean Physiological Cost Index (PCI) and lower gait speed than four-wheeled walkers, suggesting that walker configuration may influence functional walking performance in older adults [12]. Moreover, lateral falls during turning were more frequent among 2-wheeled walker users, while 4-wheeled walker users experienced more backward falls during weight shifting [13]. These incidents highlight the persistent risk of falls even when assistive devices are used, often due to device limitations such as poor maneuverability and inadequate lateral stability. Advanced walker designs have sought to address these challenges. An instrumented 4-wheeled rollator incorporating load cells, pressure-sensing insoles, and 3D motion capture was developed to objectively assess user stability and device loading during different walking scenarios. These findings underscore the need for assistive technologies that can accommodate the complex mobility demands encountered by older adult users [14].

Although previous studies have reported the development and technical capabilities of smart walker technologies, evidence regarding their effects on walking efficiency, dynamic balance, physiological responses, and fear of falling in community-dwelling older adults remains limited [15,16]. Moreover, few randomized crossover studies have directly compared a smart wheeled walker with a conventional wheeled walker under supervised clinical conditions [16]. To address these knowledge gaps, we developed a novel smart wheeled walker integrating safety features, ergonomic adjustability, and physiological monitoring capabilities. The smart wheeled walker integrates 4 complementary functional components. First, the ergonomic frame, height-adjustable handles, freely rotating wheels, and braking mechanism were designed to improve maneuverability, walking efficiency, and dynamic balance. Second, the integrated HR and SpO_₂_ monitoring system provides real-time physiological information to support assessment of the physiological cost of walking and cardiopulmonary responses during ambulation. Third, the tilt sensor–based fall detection system and mobile alert function enhance safety monitoring by notifying caregivers when excessive tilt or a fall is detected. Finally, the foldable and portable design was intended to facilitate transportation and storage of the device. These design features may support future implementation of smart mobility technologies, although their real-world usability and acceptability were not evaluated in the present study. HR monitoring is particularly relevant to the present study because the PCI, the primary outcome measure of walking efficiency, is calculated from the difference between walking HR and resting HR divided by walking speed. Consequently, continuous HR monitoring provides the physiological data required for objective estimation of the physiological cost of walking during functional ambulation [17]. In addition, SpO_₂_ monitoring serves as an adjunct physiological safety indicator for monitoring cardiopulmonary responses during walking. These monitoring functions support physiological assessment and safety monitoring but are not intended to directly improve walking efficiency, dynamic balance, or fear of falling. Rather, improvements in walking efficiency and dynamic balance are more likely attributable to the walker’s ergonomic design, height adjustability, maneuverability, and braking mechanism, while the physiological monitoring and fall detection functions provide additional clinical information to support safer mobility assessment and use [15-17]. Previous studies have demonstrated the feasibility of smart walker technologies and physiological monitoring systems in supporting mobility assessment among older adults. However, these studies evaluated different walker designs, sensor systems, walking tasks, and outcome measures, making direct comparisons difficult. Despite these promising technological advances, high-quality comparative clinical evidence evaluating the effectiveness of smart wheeled walkers remains limited, particularly randomized crossover studies assessing both functional and physiological outcomes under standardized supervised conditions.

Therefore, this randomized crossover trial aimed to compare walking efficiency, dynamic balance, physiological responses, and fear of falling between a smart wheeled walker with integrated physiological monitoring and fall detection and a standard wheeled walker during supervised use among community-dwelling older adults.

## Methods

### Development Process of the Smart Wheeled Walker

#### Step 1: Structural and Ergonomic Design

##### Smart Wheeled Walker

The smart wheeled walker was engineered with a lightweight aluminum alloy frame to provide structural stability while minimizing the physical effort required during ambulation. The device weighed approximately 6.9 kg and supported users weighing up to 120 kg. It featured a compact foldable design (51 cm × 59.6 cm × 86.6 cm) and 4 multidirectional wheels (2 front and 2 rear) to enhance maneuverability during walking. The frame was constructed from SUS-304 stainless steel hollow tubing (1-inch diameter, 1 mm thickness) to ensure durability while maintaining a lightweight structure. The handle height was adjustable to three levels (78‐88 cm), allowing users to maintain approximately 20°-30° of elbow flexion during ambulation, an ergonomic position associated with reduced upper-limb loading and improved walking efficiency. The walker incorporated a foam-cushioned seat with a backrest and underseat storage to enhance user comfort during rest periods. Dual hand-operated brakes provided both speed control during walking and secure wheel locking while seated. These structural and ergonomic features were designed to provide mechanical characteristics comparable to those of the standard wheeled walker used in this study while incorporating additional physiological monitoring and fall detection functions.

##### Standard Wheeled Walker (Comparator)

The comparator was a commercially available 4-wheeled rollator with a lightweight aluminum alloy frame. The device weighed 7.2 kg and supported users weighing up to 100 kg. Its overall dimensions were 61 cm × 70 cm × 89 cm when unfolded and 61 cm × 40 cm × 104 cm when folded. The walker was equipped with four 8-inch polyvinyl chloride (PVC) wheels, adjustable-height handles (78‐89 cm), and a foldable frame to facilitate transportation and storage. The seating system consisted of a foam-cushioned seat (35 × 32 cm) with a backrest to provide resting support during ambulation. A dual hand-operated braking system enabled speed control during walking and wheel locking while seated. The standard wheeled walker was selected because its fundamental mechanical characteristics, including the lightweight frame, 4-wheel configuration, adjustable handle height, braking mechanism, foldable design, and seating structure, were comparable to those of the smart wheeled walker. Unlike the smart wheeled walker, the standard device did not incorporate real-time physiological monitoring, fall detection, or mobile alert functions.

### Step 2: Integration of Smart Fall Detection and Alert System

The walker is embedded with a high-precision tilt sensor (accuracy ±1.5°) capable of detecting deviations in real time. When tilt exceeds 30°—an angle validated in the fall biomechanics literature as indicative of instability—a 2-tier alert protocol is activated:

1. A 90 dB audible alarm is emitted to draw attention from nearby individuals.
2. A mobile notification system transmits alerts via SMS and the LINE app to preregistered caregivers or family members.

This system was validated in a lab-controlled environment using a standardized mannequin drop test, replicated across multiple angles and surfaces. Sensor reliability was cross-verified with external motion capture systems (Vicon, Oxford Metrics) and achieved 96% detection accuracy across 30 trials.

### Step 3: Physiological Monitoring Module

To assess exertional load, the walker integrates a commercially calibrated photoplethysmography (PPG) sensor and a pulse oximeter (certified ISO 80601-2-61). The system continuously monitors:

- HR (beats per minute [bpm])
- Peripheral SpO_₂_ (%)

Real-time physiological data are displayed on a 2.4-inch OLED (organic light-emitting diode) interface in large, high-contrast fonts, allowing immediate feedback for users and clinicians. Sensor accuracy was benchmarked against a validated fingertip pulse oximeter (Masimo Radical-7) and showed a mean absolute error of less than ±2 bpm (HR) and ±1.8% (SpO_₂_), consistent with clinical-grade standards.

All instrumentation underwent repeated calibration and testing in both static and dynamic conditions (eg, walking on a treadmill at 1.0‐1.5 m/s). A pilot usability study (n=10 older adults) confirmed signal stability during typical community-based ambulation.

### Step 4: Testing of the Developed Smart Wheel Walker

#### Participants

This study included 30 community-dwelling older adults aged between 65 and 80 years, residing in Chonburi Province, Thailand. Participants were recruited from local communities in Chonburi Province, Thailand, through community announcements, village health volunteers, and referrals from local health care personnel. Individuals who expressed interest were invited to the Physical Therapy Laboratory, Burapha University, where eligibility screening was conducted according to the predefined inclusion and exclusion criteria. Eligible participants received verbal and written information about the study, provided written informed consent, and were subsequently enrolled in the trial. Eligibility criteria required that participants be capable of walking independently and performing activities of daily living without assistance. To ensure a mild balance deficit, the Timed Up and Go (TUG) test was administered, and only individuals with scores greater than 13.5 seconds were enrolled. Exclusion criteria included a diagnosis of any neurological condition that impaired gait and orthopedic or musculoskeletal surgery of the upper or lower limbs within the past 3 months. Additional exclusions were uncorrectable visual impairments such as cataracts or diabetic retinopathy and any severe cardiovascular or musculoskeletal disorders that could interfere with safe ambulation. The study adhered to ethical guidelines, including the right to withdraw at any time and the protection of participant confidentiality. Following enrollment, participants were assigned to the intervention sequence in this randomized crossover trial. After completing the first walking condition, participants rested for 30 minutes to minimize carryover effects before using the alternate walker. The order of walker use was counterbalanced across participants to control for potential sequencing bias.

#### Study Design

This study used a single-blind randomized crossover trial design in which the outcome assessor was blinded to the allocation sequence. Randomization was performed by an independent researcher using a computer-generated random allocation sequence created with the RAND function in Microsoft Excel. Allocation concealment was maintained using sequentially numbered, sealed, opaque envelopes. Outcome assessments were conducted by a separate blinded assessor who was not involved in the randomization process or intervention administration. Because of the visible differences between the smart wheeled walker and the standard wheeled walker, participant blinding was not feasible. Participants were randomly assigned to begin with either the smart wheeled walker or the standard wheeled walker. After completing the first intervention, participants crossed over to the alternate walker following a 30-minute washout period to minimize potential carryover effects. The washout period was selected to allow physiological recovery following the brief 20-m walking task, particularly recovery of HR before the second assessment. Before formal testing, all participants completed a familiarization session with both walkers. Standardized instructions on the correct use of each device were provided, and participants practiced walking with both walkers before outcome measurements were performed. The short walking distance and self-selected comfortable walking speed were considered unlikely to induce substantial fatigue or persistent physiological effects that could influence the subsequent assessment. This randomized crossover trial was designed to compare the short-term effects of a smart wheeled walker and a standard wheeled walker under standardized, supervised clinical conditions. The primary objective was to evaluate walking efficiency using the PCI. Secondary objectives were to assess dynamic balance and fear of falling. HR and walking speed were recorded to support the calculation and interpretation of PCI rather than as independent confirmatory outcomes. This study was not intended to evaluate long-term clinical effectiveness, independent community use, or real-world implementation. The study was reported in accordance with the CONSORT-eHEALTH (Consolidated Standards of Reporting Trials of Electronic and Mobile Health Applications and Online Telehealth) checklist (Checklist 1).

### Ethical Considerations

This study was approved by the Burapha University Research and Innovation Administration Committee (IRB1-115/2567). The trial was prospectively registered in the Thai Clinical Trials Registry (TCTR20250302008). The study conformed to the ethical standards set forth in the Declaration of Helsinki. All data collection was conducted between April and May 2025 at the Physical Therapy Laboratory of Burapha University, Thailand. Written informed consent was obtained from all participants before enrollment. Participants were informed that participation was voluntary and that they could withdraw from the study at any time without consequences. All data were deidentified before analysis and stored securely to maintain participant confidentiality. Participants received reimbursement for travel expenses but did not receive any additional financial compensation or incentives for participation.

### Sample Size Estimation

The sample size was determined using G*Power version 3.1.9.2. The calculation was based on the primary outcome (PCI). An effect size of 0.60 was estimated from preliminary paired PCI data obtained from 10 community-dwelling older adults who completed walking assessments using both the smart wheeled walker and the standard wheeled walker. Assuming a 2-tailed paired *t* test, a significance level (α) of .05, and a statistical power (1−β) of .80, the estimated minimum sample size was 24 participants. To account for an anticipated 20% attrition rate, the target sample size was increased to 30 participants.

### Experimental Procedure

Participants attended an orientation session 1 day prior to testing at the Faculty of Allied Health Sciences, Burapha University. The procedures were explained, and participants practiced adjusting walker height to the level of the greater trochanter, maintaining elbow flexion between 20° and 30°. They were also familiarized with walking using both a standard wheel walker and a smart wheeled walker.

On the testing day, participants were fitted with a Polar FT7 HR monitor (Polar Electro Oy). Resting HR was recorded over 5 minutes before walking trials. Participants then walked 20 m at a normal pace using one of the 2 walker types, with HR recorded every 30 seconds during walking and a 5-minute seated recovery period. Walking speed was also measured. After a 30-minute rest, participants repeated the protocol with the alternate walker. Walker order was randomized.

### Outcome Measurements

#### Primary Outcome: Walking Efficiency

Walking efficiency was quantified using the PCI, calculated as follows: PCI (beats/m) = (HR during walking – Resting HR)/Average walking speed, where HR is expressed in bpm and average walking speed in meters per minute (m/min). A lower PCI value reflects a more efficient gait pattern. This metric was applied to compare energy expenditure during ambulation with both the smart-wheeled walker and the standard walker, thereby reflecting the physiological cost of walking [18]. The PCI was the prespecified primary outcome and served as the principal confirmatory end point. HR and walking speed were measured to support the calculation and physiological interpretation of PCI and were not considered independent confirmatory outcomes. Dynamic balance (Expanded Timed Up and Go [ETUG]) and fear of falling (Falls Efficacy Scale-International [FES-I]) were evaluated as prespecified secondary outcomes.

#### Secondary Outcomes

##### Dynamic Balance

Dynamic balance was evaluated using the ETUG test.

Participants were instructed to rise from a chair, walk a 20-m distance using either walker, circle a designated marker, and return to sit. The time to complete the task was recorded in seconds [19].

##### Fear of Falling

Fear of falling was assessed using the Thai version of the FES-I, which was adapted and validated by Thiamwong based on the original instrument developed by the Prevention of Falls Network Europe (ProFaNE) [20]. The scale includes 16 items that assess concern about falling during a range of physical and social activities, from basic daily tasks to more demanding functions. Participants rated their concern on a 4-point Likert scale: 1 (not at all concerned), 2 (somewhat concerned), 3 (fairly concerned), and 4 (very concerned). Total scores range from 16 to 64. Scores of 16 to 21 indicate low concern about falling, 22 to 27 reflect moderate concern, and 28 to 64 represent high concern. This scale has been widely used to evaluate fall-related self-efficacy among older adults in community and clinical settings.

### Data Analysis

All statistical analyses were conducted using SPSS (SPSS Inc.). Descriptive statistics were used to summarize demographic data. The Shapiro-Wilk test was used to assess the normality of outcome variables. Paired *t* tests were used to compare resting HR, walking HR, average walking speed, PCI, ETUG performance, and fear of falling between the 2 walker conditions for normally distributed data. In addition, a repeated-measures ANOVA was performed as a supplementary analysis for the randomized crossover design. The model included walker condition (smart wheeled walker vs standard wheeled walker) and period (first vs second assessment) as within-participant factors and sequence (smart-standard vs standard-smart) as a between-participant factor, with participants treated as the repeated subject. Sequence and period effects were examined to indirectly assess potential residual carryover effects. Because of the relatively small sample size, these analyses were considered exploratory and were interpreted with caution. A 2-sided *P* value of <.05 was considered statistically significant.

## Results

### Overview

**Figure 1.**
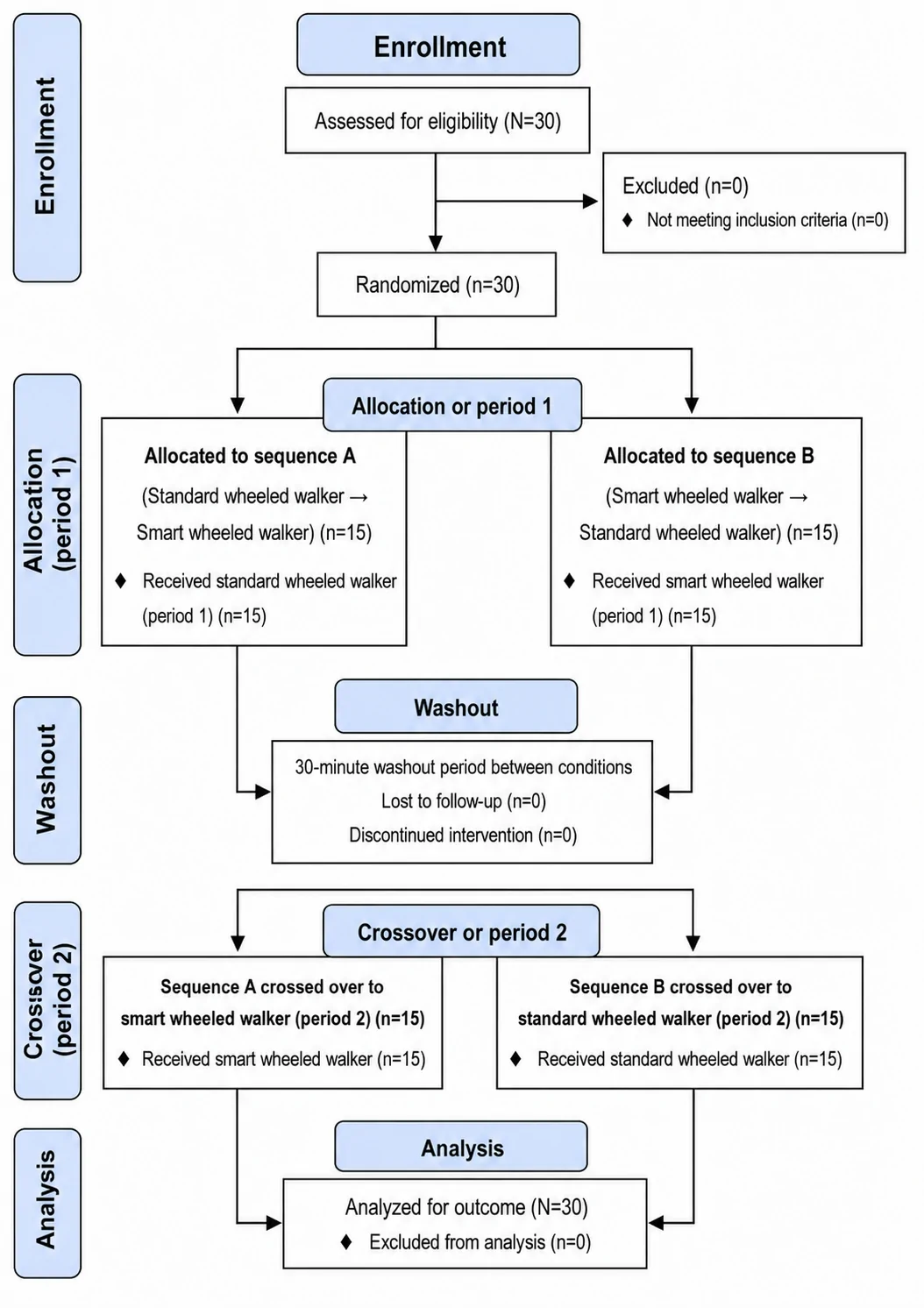
CONSORT (Consolidated Standards of Reporting Trials) 2010 flow diagram of participant enrollment, randomization, allocation to intervention sequence, crossover, follow-up, and analysis.

Figure 1 presents the CONSORT 2010 flow diagram illustrating the flow of participants through enrollment, randomization, allocation to intervention sequence, crossover, follow-up, and analysis. A total of 30 community-dwelling older adults were enrolled and completed both intervention conditions. No participants were lost to follow-up or excluded from the final analysis.

### Participant Characteristics

Thirty older adults participated in the study, including 7 (23.33%) men and 23 (76.67%) women. The mean age was 69.83 (SD 3.51) years. The average height and weight were 158.40 (SD 8.29) cm and 60.41(SD 10.99) kg, respectively, yielding a mean BMI of 24.20 (SD 3.56) kg/m². Functional mobility was assessed using the ETUG test, with an average completion time of 13.98 (SD 0.85) seconds (Table 1).

**Table 1.** Baseline characteristics of the participants (N=30).

Variable	Values
Sex, n (%)	
Male	7 (23.33)
Female	23 (76.67)
Age (y), mean (SD)	69.83 (3.51)
Weight (kg), mean (SD)	60.41 (10.99)
Height (cm), mean (SD)	158.40 (8.29)
BMI (kg/m²), mean (SD)	24.20 (3.56)
ETUG^a^ test (3 m, seconds), mean (SD)	13.98 (0.85)

a ETUG: Expanded Timed Up and Go.

### Primary Outcome: Walking Efficiency

The analysis showed that HR measurements taken before (*P*=.92), during (*P*=.55), and after walking (*P*>.99) did not differ significantly between the smart wheeled walker and standard wheel walker conditions. However, participants demonstrated a significantly faster average walking speed over the 20-m course when using the smart wheeled walker (*P*<.001). In addition, the PCI, which reflects the energy cost of walking, was significantly lower in the smart wheeled walker condition compared to the standard wheel walker (*P*=.02), indicating more efficient ambulation with the smart device.

### Secondary Outcomes

#### Dynamic Balance

Dynamic balance, evaluated through the ETUG test, showed a statistically significant improvement when participants used the smart wheeled walker. The time required to complete the TUG test was notably shorter with the smart device than with the standard walker (*P*=.048), indicating better balance and mobility performance.

#### Fear of Falling

The findings of this study indicated that participants who walked with the smart wheeled walker reported lower levels of fear of falling compared to those who used the standard wheel walker. However, the difference between the 2 groups did not reach statistical significance (*P*=.62; Table 2).

**Table 2.** Comparison of heart rate (HR), walking efficiency, average speed, and dynamic balance between standard walker and smart wheel walker.

Variables	Standard wheel walker (n=30), mean (SD)	Smart wheel walker (n=30), mean (SD)	Difference among groups (95% CI)	*P^a^* value
Walking efficiency (PCI^b^, beats/m)	0.69 (1.23)	0.36 (0.38^c^)	0.33 (0.06 to 0.60)	.02
Resting HR (beats/minute)	71.72 (11.36)	71.35 (11.80)	0.37 (−5.61 to 6.35)	.92
Walking HR (beats/minute)	85.47 (11.34)	83.47 (11.89)	2.00 (−4.00 to 8.00)	.55
Postwalking HR (beats/min)	73.23 (12.01)	73.20 (12.62)	0.03 (−6.33 to 6.39)	>.99
Average walking speed (20 m, m/min)	18.14 (14.55)	28.77 (11.94^c^)	−10.63 (−17.51 to −3.75)	<.001
Dynamic balance (ETUG^d^, seconds)	64.47 (23.04)	50.06 (24.50^c^)	14.41 (2.12 to 26.70)	.048
Fear of falling (scores)	32.91 (10.37)	31.56 (9.58)	1.35 (−3.81 to 6.51)	.62

a *P*<.05 was considered statistically significant.

b PCI: Physiological Cost Index.

c Significantly different between walker types.

d ETUG: Expanded Time Up and Go.

Additional crossover analyses demonstrated no significant sequence effects for PCI (*P*=.62), ETUG (*P*=.54), walking speed (*P*=.71), or FES-I (*P*=.83). Similarly, no significant period effects were observed for any study outcome (all *P*>.05). These findings indicate that the order of walker administration and testing period did not materially influence the treatment comparisons between the smart wheeled walker and the standard wheeled walker.

## Discussion

### Principal Findings

This study investigated the comparative impact of a smart wheeled walker and a standard walker on various aspects of mobility and user experience in older adults. The smart wheeled walker, which incorporates advanced functions, including adjustable height, multidirectional wheels, biometric feedback, and real-time fall alert capabilities, was associated with favorable short-term outcomes in several mobility-related measures under supervised testing conditions.

The smart wheeled walker was associated with improved walking efficiency, as reflected by a significantly lower PCI under the supervised conditions of the present study (0.36 beats/m), compared with the standard wheeled walker (0.69 beats/m). This value was lower than that previously reported for conventional wheeled walkers (1.23 beats/m) [12], although direct comparisons between studies should be interpreted with caution because of differences in study populations and experimental protocols. This improvement is likely attributable to multiple design features aimed at optimizing mechanical advantage and reducing muscular workload. The device’s adjustable height mechanism allowed users to maintain an elbow flexion angle of approximately 30°, a position that supports optimal force transfer through the upper extremities during assisted gait. This alignment may have minimized unnecessary recruitment of the shoulder depressor muscles, such as the latissimus dorsi and triceps brachii, particularly in taller individuals [2], resulting in reduced fatigue and more efficient propulsion. Additionally, the multidirectional wheels and lightweight aluminum frame likely contributed to smoother gait cycles by decreasing friction and minimizing the need for abrupt deceleration or manual lifting of the device. These factors together may have reduced the cumulative metabolic demand during ambulation. Although the mean HR during walking was not statistically different between the groups, participants using the smart wheeled walker exhibited slightly lower average HRs (83.47, SD 11.89 bpm vs 85.47, SD 11.34 bpm). This trend may reflect a lower cardiovascular load, secondary to the improved mechanical efficiency and reduced physical exertion required to maneuver the device. These physiological findings are in line with prior evidence demonstrating that walker design can influence energy expenditure and cardiopulmonary response during walking [1].

The results of this study suggest that participants demonstrated better dynamic balance while using the smart wheeled walker than the standard wheeled walker, as reflected by ETUG performance under supervised testing conditions. Participants using the smart wheeled walker completed the test in less time (50.06, SD 24.50 s) than those using the standard wheeled walker (64.47, SD 23.04 s), suggesting superior walking efficiency and postural control. These findings align with previous research that demonstrated that wheeled walkers can reduce energy expenditure and support faster straight-line walking in older adults [9]. Previous studies have also reported that wheeled walkers improve balance and stability during ambulation [6]. However, the present study did not evaluate fall incidence; therefore, the potential effect on fall prevention remains to be confirmed in future longitudinal studies [6]. The current study findings are also consistent with previous evidence suggesting that wheeled walkers help prevent physical decline by enabling safe and sustained physical activity in older individuals. Importantly, the smart wheeled walker used in this study featured a height-adjustable frame with 3 levels, allowing users to begin walking from an ergonomically optimal elbow flexion angle of approximately 30° [21]. This positioning reduces arm fatigue and supports more natural and stable movement. Previous research has also demonstrated that height-adjustable walkers are associated with improved balance during ambulation [22]. Additionally, the smart wheeled walker incorporated front and rear wheel locking mechanisms, enhancing safety during use. These braking systems likely provided users with greater control during transitions and when navigating turns or uneven movements. These braking systems may have contributed to improved maneuverability and perceived stability during walking tasks, which may contribute to safer mobility during supervised walking tasks. The potential effect on fall prevention requires confirmation in longitudinal studies. Taken together, these findings suggest that the smart wheeled walker may have the potential to support mobility in older adults under supervised conditions. Because ETUG was a prespecified secondary outcome and no formal adjustment for multiple comparisons was performed, these findings should be interpreted as supportive rather than confirmatory evidence. Therefore, larger randomized controlled trials with long-term follow-up are required to confirm these findings.

In this study, the average score on the fear of falling scale was slightly lower among participants using the smart wheeled walker compared to those using the standard walker, although the difference was not statistically significant. However, the numerical trend observed in the smart wheeled walker condition may warrant further investigation in studies with larger sample sizes and longer follow-up. The smart walker included features such as a lightweight aluminum frame, adjustable handles, 4 rotatable wheels, a built-in seat, and a dual-braking system. Furthermore, it incorporated intelligent capabilities including a tilt sensor that detected deviations greater than 30° and transmitted alerts through mobile messaging apps. The integrated display of real-time HR and SpO_₂_ may also have provided additional reassurance to users and caregivers. These design features likely enhanced the sense of psychological security, particularly in individuals who ambulate independently or reside alone. Previous studies have suggested that automatic fall detection systems and health-monitoring functions may reduce anxiety related to safety. However, the present study did not evaluate the real-world performance of the fall detection and alert system during actual fall events. Previous research has highlighted the association between elevated fear of falling and decreased physical and social engagement, which may in turn increase the risk of functional decline and actual falls [23]. Although the difference was not statistically significant, the numerical trend observed in the present study may warrant further investigation in adequately powered studies with longer follow-up to determine whether the smart wheeled walker has a meaningful effect on fear of falling, particularly among older adults at increased risk of falls.

This study has several important limitations. First, the inclusion criteria did not exclude participants with chronic conditions or those taking medications that could influence cardiovascular responses, such as beta-blockers. These factors may have affected HR data and walking performance outcomes. Future research should consider refining the exclusion criteria to control for medical conditions and pharmacological influences that could confound physiological measurements. Second, the number of previous falls among participants was not documented. Since fall history is a known predictor of mobility confidence and functional balance, its absence limits the ability to interpret fear of falling and balance-related outcomes fully. Third, the study was conducted in a controlled indoor setting with uniform flooring. This limits ecological validity, as real-world environments often include uneven surfaces, curbs, or visual distractions that challenge gait and balance. Future studies should assess device performance across diverse environmental conditions to better reflect everyday use. Fourth, no measures of oxygen consumption or electromyographic activity were included. Incorporating such physiological metrics would allow for a more comprehensive understanding of energy expenditure and muscle activation patterns during device use. Fifth, the gender distribution in this sample was notably imbalanced, with a higher number of female participants. Given known sex-related differences in gait characteristics, strength, and perceptions of fall risk, this imbalance may have influenced the study’s findings. Future studies should strive for more balanced recruitment or conduct stratified analyses to better understand sex-specific responses to assistive technologies. Sixth, the smart wheeled walker and the standard wheeled walker differed not only in their smart monitoring and fall alert functions but also in several mechanical and ergonomic characteristics, including frame design, wheel configuration, and adjustability. Therefore, the observed differences between the 2 devices cannot be attributed solely to the smart monitoring and alert functions, as the mechanical and ergonomic differences between the devices may also have influenced walking performance and user outcomes. In addition, although the smart wheeled walker incorporates fall detection and mobile alert functions, the present study did not evaluate the real-world performance of these features during actual fall events or independent community use. Although the smart wheeled walker demonstrated promising short-term benefits under supervised conditions, several factors should be considered before its implementation in real-world settings. Older adults may differ in digital literacy, cognitive and sensory abilities, and their willingness to use technology-assisted devices. Caregiver support may be necessary for some users, particularly when responding to remote alerts or assisting with device operation. In addition, privacy and data security should be carefully addressed when transmitting health-related information. Future studies should evaluate the long-term usability, acceptability, safety, and implementation of the smart wheeled walker during independent home and community use in more diverse older adult populations. In addition, the present study did not systematically collect data on usability, user experience, or comprehensive safety outcomes, including adverse events during prolonged use. These aspects should be incorporated into future studies to provide a more comprehensive evaluation of the smart wheeled walker. Seventh, the study assessed only short-term use of each walker type. Longitudinal follow-up would be valuable for evaluating sustained effects on physical performance, fall incidence, and user adherence over time. The randomized crossover design enabled each participant to serve as his or her own control, thereby reducing interindividual variability. Additional crossover analyses demonstrated no significant sequence or period effects, suggesting that the order of walker administration and testing period did not materially influence the study outcomes. However, because of the relatively small sample size, these findings should be interpreted with caution and do not completely exclude the possibility of residual sequence or carryover effects. Moreover, participant familiarization before testing, randomized and counterbalanced allocation, and a 30-minute washout period were implemented to minimize potential carryover, learning, and fatigue effects. Nevertheless, because the study was conducted during a single supervised testing session, residual carryover or learning effects cannot be completely excluded. Future crossover studies with larger sample sizes and longer follow-up should further evaluate these potential influences. In addition, the generalizability of the present findings should be interpreted with caution. The study included only community-dwelling older adults aged 65 to 80 years, with a predominance of female participants, and all assessments were conducted under supervised laboratory conditions. Therefore, the findings may not be generalizable to frailer older adults, institutionalized individuals, people with cognitive impairment, or unsupervised community settings. Future studies should include more diverse populations and evaluate the performance of smart wheeled walkers in real-world environments to improve the external validity of the findings. Finally, this randomized crossover trial involved a relatively small sample size and a single supervised testing session. Therefore, the findings should be interpreted as evidence of short-term performance under supervised conditions rather than definitive evidence of long-term clinical effectiveness or real-world functional benefits. Future adequately powered randomized controlled trials with extended follow-up in community settings are warranted to confirm these findings and evaluate the long-term effectiveness, safety, and user adherence of the smart wheeled walker.

### Conclusions

This randomized crossover clinical trial showed that the smart wheeled walker was associated with improved walking efficiency and dynamic balance under supervised testing conditions when compared with a standard wheeled walker. Cardiovascular responses and fear of falling did not differ significantly between walker conditions. Because the smart wheeled walker was evaluated as an integrated device, the independent contribution of individual design features could not be determined. Further randomized controlled trials with larger and more diverse samples, long-term follow-up, and real-world evaluation are needed to evaluate the individual and combined effects of specific design features and to further establish the clinical effectiveness and practical applicability of smart wheeled walkers for older adults.

## Supplementary material

10.2196/99522 Checklist 1 CONSORT-eHEALTH checklist.

## Abbreviations

bpm: beats per minute
CONSORT-eHEALTH: Consolidated Standards of Reporting Trials of Electronic and Mobile Health Applications and Online Telehealth
ETUG: Expanded Timed Up and Go
FES-I: Falls Efficacy Scale-International
HR: heart rate
OLED: organic light-emitting diode
PCI: Physiological Cost Index
PPG: photoplethysmography
ProFaNE: Prevention of Falls Network Europe
PVC: polyvinyl chloride
SpO_2_: oxygen saturation
TUG: Timed Up and Go
